# In Vitro Activities of Colistin and Sitafloxacin Combinations against Multidrug-, Carbapenem-, and Colistin-Resistant *Acinetobacter baumannii* Using the Broth Microdilution Checkerboard and Time-Kill Methods

**DOI:** 10.3390/antibiotics9080516

**Published:** 2020-08-14

**Authors:** Vipavee Rodjun, Jantana Houngsaitong, Preecha Montakantikul, Taniya Paiboonvong, Piyatip Khuntayaporn, Pattareeya Yanyongchaikit, Pusana Sriyant

**Affiliations:** 1 Faculty of Pharmacy, Siam University, Bangkok 10160, Thailand; vipavee.rod@siam.edu; 2 Faculty of Pharmacy, Mahidol University, Bangkok 10400, Thailand; preecha.mon@mahidol.ac.th (P.M.); piyatip.khn@mahidol.ac.th (P.K.); pattareeyapatz@gmail.com (P.Y.); nistaarm.psn@gmail.com (P.S.); 3 Faculty of Pharmacy, Rangsit University, Pathum Thani 12000, Thailand; taniya.p@rsu.ac.th

**Keywords:** polymyxin, fluoroquinolone, multidrug-resistant Gram-negative bacteria, carbapenem-resistant Gram-negative bacteria, colistin-resistant Gram-negative bacteria, combination, synergy, synergistic effect

## Abstract

Drug-resistant *Acinetobacter baumannii (A. baumannii*) infections are a critical global problem, with limited treatment choices. This study aims to determine the in vitro activities of colistin–sitafloxacin combinations against multidrug-, carbapenem- and colistin-resistant *A. baumannii* (MDR-AB, CRAB, CoR-AB, respectively) clinical isolates from tertiary care hospitals. We used the broth microdilution checkerboard and time-kill methods in this study. Synergy was found using both methods. The colistin–sitafloxacin combination showed synergy in MDR-AB, CRAB, and CoR-AB isolates (3.4%, 3.1%, and 20.9%, respectively). No antagonism was found in any type of drug-resistant isolate. The majority of CoR-AB isolates became susceptible to colistin (95.4%). The time-kill method also showed that this combination could suppress regrowth back to the initial inocula of all representative isolates. Our results demonstrated that the colistin–sitafloxacin combination might be an interesting option for the treatment of drug-resistant *A. baumannii*. However, further in vivo and clinical studies are required.

## 1. Introduction

*Acinetobacter baumannii* (*A. baumannii*) is one of the most common causes of nosocomial pneumonia in Asia [1]. Currently, several types of resistance in *Acinetobacter* spp. are critical problems worldwide, including MDR-AB (multidrug-resistant *A. baumannii*), CRAB (carbapenem- resistant *A. baumannii*), and CoR-AB (colistin-resistant *A. baumannii*), which result in long hospitalizations and high mortality and morbidity rates [2,3]. This Gram-negative bacterium has various mechanisms of antibiotic resistance. For example, the mechanism behind its resistance to beta-lactams, including carbapenems, includes the production of beta-lactamases or changes in the outer membrane proteins and multidrug efflux pumps [4]. The mechanism of resistance to fluoroquinolones involves target mutation and efflux pump over-activity [4]. For aminoglycosides, drug-inactivating enzymes, target mutation, and efflux pump over-activity are the mechanisms of resistance [4]. Multidrug-resistant *A. baumannii* involves many complex mechanisms and more than 40 different genes [5].

Colistin is a polymyxins antibiotic agent [6] that is commonly used in both monotherapy and combination therapy for the treatment of infections caused by drug-resistant Gram-negative bacteria [7,8]. Colistin-based combination regimens have shown superior clinical outcomes or even microbiological eradication compared to colistin alone [9,10]. However, some trials have shown no significant differences between colistin monotherapy and combination therapy in clinical outcomes [11,12]. Thus, colistin-based combination therapy has been controversial in terms of results, depending on the drug that is combined with colistin and the types of infections or pathogens being targeted. Despite this, colistin remains a drug of choice for Gram-negative bacterial infections. Unfortunately, worldwide, the problem of CoR-AB has increased every year since its emergence [13,14]. The mechanism of resistance to polymyxins is target mutation, such as decreased stability of the outer membrane [4]. Several in vitro studies have been conducted using various antibiotic class combinations against *A. baumannii* and various methods, such as fosfomycin-imipenem by the checkerboard method and imipenem-sulbactam by the time-kill method, which have shown diverse results [15]. Several in vivo and in vitro studies of colistin-based regimens against MDR-, XDR- (extensively drug-resistant), and CRAB showed a synergistic effect [16,17,18,19,20,21,22,23,24]. The colistin-fluoroquinolones combination was one of many that showed a strong synergistic effect in these studies [17,23,24]. Sitafloxacin is a new fluoroquinolone with proven in vitro activity against a broad range of Gram-positive and Gram-negative bacteria, including anaerobic bacteria, and also against atypical pathogens [25]. It has shown synergy against XDR-AB when combined with rifampin, sulbactam, and colistin [24,26]. No prior studies have discovered the efficacy of sitafloxacin combinations in other types of drug-resistance, such as multidrug-resistance, carbapenem-resistance, and colistin-resistance in *A. baumannii*. To date, there have been limited choices for the treatment of drug-resistant *A. baumannii*. We conducted this study to investigate the synergistic activities of colistin in combination with sitafloxacin against MDR-AB, CRAB, and CoR-AB, the results of which might be helpful for further clinical studies.

## 2. Results

### 2.1. Susceptibility and Minimum Inhibitory Concentration (MIC) Testing

In total, 300 *A. baumannii* isolates were isolated from sputum (233 isolates), pus (28 isolates), urine (16 isolates), blood (13 isolates), tissue (6 isolates), peritoneal dialysis fluid (2 isolates), bone tissue (1 isolate), and a nasal swab (1 isolate). The resistant isolates were composed of MDR-AB 263 isolates (87.7%), CRAB 258 isolates (86%), and CoR-AB 43 isolates (14.3%). There were 253 CRAB isolates (96.2%) among the MDR-AB isolates.

The minimum inhibitory concentration (MIC) values of all isolates were tested by the broth microdilution method. The MIC range of colistin was 0.5–16 milligrams per liter (mg/L) in the MDR-AB and CRAB isolates and 4–16 mg/L in the CoR-AB isolates. The MIC range of sitafloxacin of the MDR-AB, CRAB, and CoR-AB isolates was 0.02–8 mg/L. We also determined the MIC of the 50th and 90th percentiles (MIC_50__/90_) of each type of isolate. The MIC_50__/90_ of colistin was 2/4 mg/L in the MDR-AB and CRAB isolates, and 8/8 mg/L in the CoR-AB isolates. The MIC_50__/90_ of sitafloxacin was 1/2 in the MDR-AB and CRAB isolates and 0.5/1 in the CoR-AB isolates. The percentages of susceptibility of colistin and sitafloxacin were calculated from the following formula: (the level of susceptibility of the isolate to each agent/the level of all isolates in each type) × 100. More than 80% were colistin-susceptible isolates among the MDR-AB and CRAB isolates, and more than 95% were sitafloxacin-susceptible isolates among all types. The MIC range, MIC_50__/90_, and the colistin and sitafloxacin percentage susceptibility of each type of isolate are shown in Table 1.

### 2.2. Synergy Testing

#### 2.2.1. Broth Microdilution Checkerboard Method

After combination, the MIC values of colistin and sitafloxacin were reduced. The MIC range of colistin was 0.06–8 mg/L in the MDR-AB and CRAB isolates and 0.05–8 mg/L in the CoR-AB isolates. The MIC range of sitafloxacin in the MDR-AB and CRAB isolates was 0.01–4 mg/L and 0.02–2 mg/L in the CoR-AB isolates. The MIC_50__/90_ values were also reduced. The MIC_50__/90_ of colistin was 0.5/1 mg/L in the MDR-AB and CRAB isolates and 1/2 mg/L in the CoR-AB isolates. The MIC_50__/90_ of sitafloxacin was 0.5/1 in the MDR-AB and CRAB isolates and 0.25/1 in the CoR-AB isolates. Moreover, some colistin- or sitafloxacin-resistant isolates became susceptible isolate. Among the CoR-AB isolates, 95.4% were susceptible to colistin. The results are shown in Table 1. The fractional inhibitory concentration index (FICI) values that were used to determine the synergistic effect in this study were calculated from the following formula: FICI = (MIC of colistin in combination/MIC of colistin alone) + (MIC of sitafloxacin in combination/MIC of sitafloxacin alone). The interpretation of the results was as follows: synergy, if FICI ≤ 0.5; no interaction, if FICI >0.5 to 4; and antagonism, if FICI >4. The colistin–sitafloxacin combination showed synergy values of 3.4% and 3.1% for the MDR-AB and CRAB isolates, respectively. For CoR-AB isolates, the combination showed a synergy of 20.9%. No antagonism was found. The distribution of the FICI values is shown in Table 2.

#### 2.2.2. Time-Kill Method

The two representatives of MDR-AB with CRAB isolates (H25, K21) were tested using the time-kill method. The first resisted sitafloxacin, whereas the second did not. No CoR-AB isolates were tested by this method. For the H25 isolate (sitafloxacin-resistant), the MICs of colistin and sitafloxacin were 1 and 8 mg/L, respectively. For the K21 isolate (sitafloxacin-susceptible), the MICs of both colistin and sitafloxacin were 2 mg/L. We aimed to find the differences between sitafloxacin-resistant and susceptible isolates with time-kill methods. Bactericidal activity was found in both isolates at 4 h when 2× the MIC of colistin was tested. In addition, 1× the MIC of sitafloxacin showed bactericidal activity in both isolates at 12 h (H25) and 4 h (K21). Colistin showed rapid bacterial regrowth at all tested concentrations.

**Figure 1:**
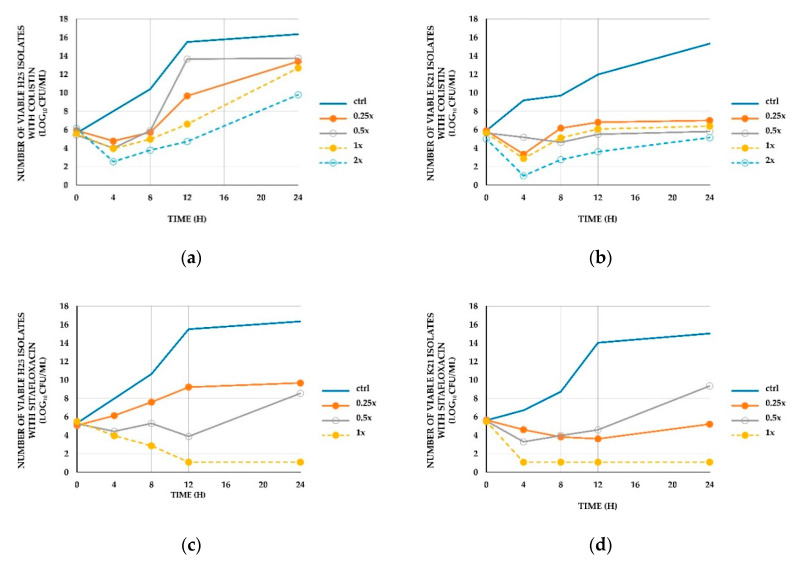
Time-kill curves of *A. baumannii* clinical isolates. Time-kill studies of (**a**) the H25 isolate with colistin, (**b**) the K21 isolate with colistin, (**c**) the H25 isolate with sitafloxacin, and (**d**) the K21 isolate with sitafloxacin. The number of viable bacteria from the start of the experiment is shown. Ctrl represents control isolates (no antimicrobial agents). The dark blue solid lines represent control isolates. The orange solid lines with filled circles represent a concentration of 0.25× MIC, the grey solid lines with open circles represent a concentration of 0.5× MIC, the yellow dashed lines with filled circles represent a concentration of 1× MIC, and the blue dashed lines with open circles represent a concentration of 2× MIC.

**Figure 2:**
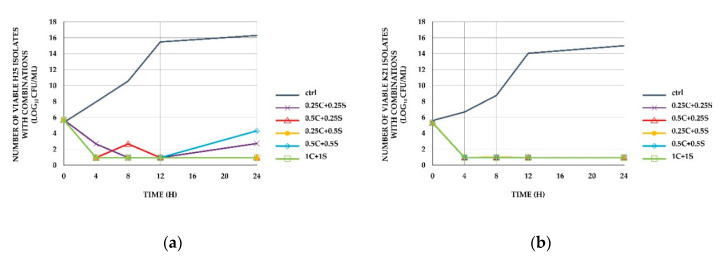
Time-kill curves of *A. baumannii* clinical isolates. (**a**) Time-kill studies of the H25 isolate with combinations, (**b**) the K21 isolate with combinations. The number of viable bacteria from the start of the experiment is shown. Ctrl represents control isolates (no antimicrobial agents). C represents the MIC of colistin as single agent. S represents the MIC of sitafloxacin as single agent. For example, 0.25C + 0.25S represents 0.25× MIC of colistin as single agent with 0.25× MIC of sitafloxacin as single agent. The blue-grey lines represent control isolates. The purple lines with crosses represent 0.25C + 0.25S, the red lines with triangles represent 0.5C + 0.25S, the yellow lines with filled circles represent 0.25C + 0.5S, the blue lines with diamonds represent 0.5C + 0.5S, and the green lines with squares represent 1C + 1S. C and S represent the MIC of colistin and sitafloxacin, respectively. We tested the same concentrations for both isolates and show all the curves in panel b. The lines of 0.25C + 0.25S, 0.5C + 0.25S, 0.25C + 0.5S, 0.5C + 0.5S, and 1C + 1S in panel b overlap.

Colistin–sitafloxacin combinations showed a bactericidal effect against both isolates at all tested concentrations. Bactericidal activity was found at 4 h. Synergy also occurred at all tested concentrations, except 1× the MIC of colistin and sitafloxacin. The lowest concentration that showed synergy was 0.25× the MIC of the colistin–sitafloxacin combination. The results of the time-kill method are shown in Figure 1 and Figure 2.

## 3. Discussion

MDR-AB and CRAB are worldwide problems that negatively impact clinical outcomes. This is the first study of the MIC distribution of *A. baumannii* from tertiary care hospitals in Thailand. Our study showed MDR-AB at 87.7%, CRAB at 86%, and CoR-AB at 14.3%. Interestingly, 96.2% of MDR-AB isolates were also CRAB.

The lack of new antimicrobial agents for use against drug-resistant organisms can lead to inadequate therapy and limited treatment options [27]. Therefore, combination regimens of antibiotics might be considered. Colistin is commonly the last resort, both singly and in combination, for the treatment of resistant Gram-negative bacteria. There have been several studies on colistin-based therapy against *A. baumannii* which showed strong and synergistic effect. Sitafloxacin has been reported to show synergy against XDR-AB when combined with rifampin, sulbactam, and colistin [24,26]. There is no prior report on the synergy of the colistin–sitafloxacin combination against MDR-AB, CRAB, and CoR-AB. Therefore, this study focused on assessing its potential synergistic activity.

The synergy of the MDR-AB and CRAB isolates with the broth microdilution checkerboard method were 3.4% and 3.1%, respectively (Table 2). A prior in vitro study on the use of colistin–sitafloxacin against XDR-*A. baumannii* by checkerboard method was carried out by Dong et al. [24]. They found a synergy of 12.5%, which is higher than our results. Because our isolates were susceptible to both colistin (86.7% and 87.2% for MDR-AB and CRAB, respectively) and sitafloxacin (96.6% and 99.5% for MDR-AB and CRAB, respectively), to a greater degree than the isolates used by Dong et al. (62.5% for colistin and 91.7% for sitafloxacin), our synergy was lower than that found by Dong et al. A study by Guelfi et al. [28], which tested polymyxin B combined with gatifloxacin against *A. baumannii* isolates, did not find any synergy. The study did not include polymyxin B- resistant isolates. Therefore, the synergy of the study could be less than in our study. We observed a reduction in the MIC values of each agent after combination (Table 1). Although the amount of MIC reduction was insufficient to show synergy in some cases, drug-resistant strains became susceptible. Our study found that more than 95% of the isolates were susceptible to sitafloxacin or colistin when tested in combination. Our study found more isolates susceptible to sitafloxacin when tested before combination compared with Xu et al. (96% and 92%, respectively) [24], who tested the sitafloxacin-sulbactam combination against XDR-AB. On the other hand, the sitafloxacin-sulbactam combination showed synergy that was higher than ours (3.4% and 16%, respectively). It was to be expected that most of our isolates that were susceptible to sitafloxacin did not affect the synergy when combined with colistin. For CoR-AB isolates, the synergy was 20.9%. Remarkably, we found that most of the CoR-AB isolates (95.4%) became colistin-susceptible isolates after testing in combination. These results showed that colistin enhanced the bactericidal activity of sitafloxacin, and vice versa. The proposed mechanism by which colistin enhanced the activity of sitafloxacin is the disruptive effect on membrane integrity, which makes the isolates more susceptible to a hydrophobic agent [29]. The logP (the partition coefficient), a particular ratio of the concentrations of a solute between a biphasic system of lipids and water, of sitafloxacin is 3.4 [30]. A positive value for logP denotes a higher concentration in the lipid phase [31]. Therefore, the logP value of sitafloxacin indicates that sitafloxacin is hydrophobic. This is the probable mechanism by which colistin enhances sitafloxacin activity. However, the mechanism by which sitafloxacin enhances the activity of colistin has not been elucidated.

The results from the time-kill method also showed synergy in representative isolates. Moreover, the results showed that colistin accelerated the bactericidal activity of sitafloxacin in sitafloxacin-resistant strains. The mechanism for this has not been fully clarified. Regarding the bactericidal effect of colistin, colistin alone showed rapid regrowth of inocula even though they were susceptible to colistin (Figure 1). We found that the combination could maintain the bactericidal effect of colistin for 24 h or could slow the regrowth so that inocula did not reach initial levels (Figure 2). This result showed that sitafloxacin supported colistin to maintain bactericidal activity. This was similar to Safarika et al.’s [22] study of the colistin-levofloxacin combination against MDR-AB using the time-kill method, whose combination suppressed the regrowth of inocula. The mechanism was unclear. A study conducted by Zhanel et al. [32] demonstrated a reduction of the MPC/MIC ratio (mutant prevention concentrations/minimal inhibitory concentration ratio) of colistin when combined with levofloxacin against *Pseudomonas aeruginosa*. This might explain our result, in which fluoroquinolones helped to suppress the mutation of the isolates.

The first limitation of this study was the lack of genotypic data of the isolates, which might have predicted the mechanism of resistance of the isolates to sitafloxacin and colistin. This could have led us to precisely propose the mechanism of the synergistic effect and to specify the type of drug-resistance that is targeted by this combination. Another limitation of this study was that we did not test the synergistic effect by the time-kill method using CoR-AB isolates. Our results indicated that synergism was rarely observed in MDR-AB and CRAB isolates but was found for CoR-AB isolates. The drug-resistant isolates became susceptible, especially CoR-AB isolates. Further studies are needed to determine if such observations are correlated with improved clinical outcomes.

## 4. Materials and Methods

### 4.1. Clinical Isolates

A total of 412 clinical isolates of *A. baumannii* were collected from patients who attended 13 tertiary care hospitals in Thailand from 2016 to 2017. The isolates were collected from non-duplicated patients. Three hundred clinical isolates of *A. baumannii* were randomly selected by counting from the first to the twenty-fifth isolate from each hospital. Regardless of drug-resistant isolates, the selected isolates included MDR-AB, CRAB, and CoR-AB isolates. MDR-AB was defined as *A. baumannii* non-susceptible to at least one agent in ≥3 antimicrobial categories, as follows; aminoglycosides, antipseudomonal fluoroquinolones, antipseudomonal carbapenems, antipseudomonal penicillin plus beta-lactamase inhibitors, folate pathway inhibitors, penicillin plus beta-lactamase inhibitors, extended-spectrum cephalosporins, polymyxins, and tetracyclines [33]. CRAB was defined as *A. baumannii* resistant to imipenem, doripenem, or meropenem, following the Clinical and Laboratory Standard Institute (CLSI) 2016 criteria for screening carbapenem-resistant strains. This study mentioned MDR in relation to CRAB isolates, meaning the MDR definition, which includes carbapenem resistance. CoR-AB was defined as *A. baumannii* resistant to colistin conforming to CLSI 2016 (the resistant breakpoint was ≥ 4 mg/L) [34]. Genotypic testing was not performed in this study.

This study was approved by The Ethics Committee of Faculty of Dentistry/Faculty of Pharmacy, Mahidol University (COA.No.MU-DT/PY-IRB 2017/040.2607).

### 4.2. Antimicrobial Agents

Colistin sulfate standard powder was purchased from Tokyo Chemical Industry, Japan. Sitafloxacin standard powder was provided by Daichi Sankyo, Thailand.

### 4.3. Synergy Testing

#### 4.3.1. Broth Microdilution Checkerboard Method

Susceptibility and MIC testing of single agents were carried out by the broth microdilution method, according to CLSI 2016. Both agents were tested and ranged from 0.015625 to 128 mg/L. The triplicate susceptibility test was incubated at 37 °C for 18 h. *Escherichia coli* ATCC25922 was used as a quality control isolate. According to CLSI 2016, susceptible and resistant breakpoints for colistin against *Acinetobacter* spp. are ≤ 2 mg/L and ≥ 4 mg/L, respectively. Breakpoints for sitafloxacin against *Acinetobacter* spp. were unavailable from CLSI 2016. Isolates with MICs of sitafloxacin ≤ 2 mg/L were tentatively considered to be susceptible to sitafloxacin, based on molecular surveillance with automated ribotyping [35].

After MIC determination of single agents for each isolate, the antibiotics for testing were prepared as 8×, 4×, 2×, 1×, 0.5×, 0.25×, and 0.125× of the MIC of each agent and the growth control. A checkerboard microdilution method was triply tested [36]. The results of the synergistic effect were determined after incubating plates at 37 °C for 18 h. The fraction inhibitory concentration index (FICI) was calculated from the sum of the fractional inhibitory concentrations according to the following formula: FICI = (MIC of colistin in combination/MIC of colistin alone) + (MIC of sitafloxacin in combination/MIC of sitafloxacin alone). The interpretation of the results was as follows: synergy, if FICI ≤ 0.5; no interaction, if FICI > 0.5 to 4; and antagonism, if FICI > 4 [37].

#### 4.3.2. Time-Kill Method

The time-kill method was used for two representative isolates with different susceptibilities to single agents. The MICs of each isolate, which were used for testing, were determined by the broth microdilution method. Colistin and sitafloxacin were tested singly and in combination and were incubated for 0, 4, 8, 12, and 24 h at 37 °C. The concentrations of colistin were 0.25×, 0.5×, 1×, and 2× MIC, whereas for sitafloxacin, the concentrations were 0.25×, 0.5×, and 1× MIC. We tested sitafloxacin at 1× MIC, as referred to in the Xu et al. study [26]. The time-kill study showed that sitafloxacin had good activity against the isolates at the concentration of 1× MIC, regardless of the susceptibility status of the isolates. The tested concentrations of this combination were concentrations of 0.25× MIC of colistin with 0.25× and 0.5× MIC of sitafloxacin, 0.5× MIC of colistin with 0.25× and 0.5× MIC of sitafloxacin, and 1× MIC of colistin with 1× MIC of sitafloxacin. Bactericidal activity was assessed as a ≥ 3 log10 reduction in colony forming unit (cfu)/mL over the time period measured, while synergy was a ≥ 2 log10 reduction in cfu/mL for the antibiotic combination in comparison with its more active constituent [38].

## 5. Conclusions

In conclusion, the combination of colistin and sitafloxacin is an interesting alternative option for the treatment of *A. baumannii* infections, including MDR-AB, CRAB, and CoR-AB infections. However, the results from this study should be further tested, which might lead to clinical studies.

## Figures and Tables

**Table 1:** Activity of colistin and sitafloxacin against *A. baumannii* as single agents and in combination.

Agent	MDR-AB (263 Isolates)						CRAB (258 Isolates)						CoR-AB (43 Isolates)					
	Alone			In Combination			Alone			In Combination			Alone			In Combination		
	MIC Range (mg/L)	MIC_50__/90_ (mg/L)	%S	MIC Range (mg/L)	MIC_50__/90_ (mg/L)	%S	MIC Range (mg/L)	MIC_50__/90_ (mg/L)	%S	MIC Range (mg/L)	MIC_50__/90_ (mg/L)	%S	MIC Range (mg/L)	MIC_50__/90_ (mg/L)	%S	MIC Range (mg/L)	MIC_50__/90_ (mg/L)	%S
colistin	0.5–16	2/4	86.7	0.06–8	0.5/1	99.6	0.5–16	2/4	87.2	0.06–8	0.5/1	99.6	4–16	8/8	0	0.05–8	1/2	95.4
sitafloxacin	0.02–8	1/2	96.6	0.01–4	0.5/1	99.2	0.02–8	1/2	96.5	0.01–4	0.5/1	99.2	0.02–8	0.5/1	95.4	0.02–2	0.25/1	100

MDR-AB, multidrug-resistant *Acinetobacter baumannii*; CRAB, carbapenem-resistant *Acinetobacter baumannii*; CoR-AB, colistin-resistant *Acinetobacter baumannii*; MIC_50__/90_, the minimum inhibitory concentration at which 50% and 90% of the isolates are inhibited; mg/L, milligrams per liter; %S, percentage susceptibility; MIC, minimal inhibitory concentration; MIC_50__/90_,the minimum inhibitory concentration of the 50th and 90th percentiles.

**Table 2:** Distribution of FICI values for the combination of colistin and sitafloxacin against *A. baumannii.*

Isolates	Synergy (FICI: ≤ 0.5)	No Interaction (FICI: > 0.5 to 4)	Antagonism (FICI: > 4)
MDR-AB (263 isolates)	3.4%	96.6%	0%
CRAB (258 isolates)	3.1%	96.9%	0%
CoR-AB (43 isolates)	20.9%	79.1%	0%

FICI, The fractional inhibitory concentration index; MDR-AB, multidrug-resistant *Acinetobacter baumannii*; CRAB, carbapenem-resistant *Acinetobacter baumannii*; CoR-AB, colistin-resistant *Acinetobacter baumannii.*
